# Noise, Hearing, and Communication in the Operating Room: A Mixed‐Methods Study

**DOI:** 10.1002/ohn.70266

**Published:** 2026-04-30

**Authors:** Sarah E. Hughes, Milisa Manojlovich, Eunice Y. Lee, Andrew S. Bolze, Candice Stegink, Dhruv Jain, Devin L. McCaslin, Kyle H. Sheetz, Rishindra M. Reddy, Michael J. Brenner

**Affiliations:** ^1^ University of Michigan Medical School Ann Arbor Michigan USA; ^2^ University of Michigan School of Nursing Ann Arbor Michigan USA; ^3^ Department of Surgery University of Michigan Ann Arbor Michigan USA; ^4^ Department of Electrical Engineering and Computer Science University of Michigan Ann Arbor Michigan USA; ^5^ Department of Otolaryngology – Head & Neck Surgery University of Michigan Ann Arbor Michigan USA

**Keywords:** acoustic stimulation, communication barriers, interprofessional relations, noise‐induced hearing loss, occupational noise, operating rooms, patient safety, surgical procedures, workplace safety

## Abstract

**Objective:**

To evaluate noise‐related communication barriers in the operating room and to identify strategies for overcoming them.

**Study Design:**

Cross‐sectional mixed‐methods survey.

**Setting:**

Tertiary academic medical center.

**Methods:**

An anonymous 21‐item electronic survey was distributed to operating room personnel, including surgeons, anesthesiologists, residents, medical students, nurses, and allied health professionals. Items assessed hearing difficulties, baseline hearing loss, communication barriers, coping strategies, and intervention preferences. Quantitative data were analyzed using descriptive statistics, chi‐square tests, and logistic regression; open‐text responses underwent thematic analysis.

**Results:**

Among 225 survey respondents 80.4% (181/225) reported difficulty hearing in the OR and 14.7% (33/225) reported difficulty every case. All respondents with suspected or confirmed hearing loss reported hearing difficulty during surgery. Respondents with ≥5 years of OR exposure had higher odds of diagnosed hearing loss versus <5 years (OR 3.0, 95% CI 1.2‐7.9; *P* = .024); there was no association by age (*P* = .89). Among hearing aid users, 88% struggled to understand speech in the OR. Communication was most challenging in orthopedic cases (31%), with environmental noise sources (suction, drills, music, alarms) frequently cited (29%), and during robotic surgery (24%). Common self‐accommodations included asking for repetition, anticipating steps, environmental adjustments, and positioning. Qualitative themes emphasized challenges arising from ambient noise, physical barriers, and psychosocial factors such as hierarchy or lack of psychological safety.

**Conclusion:**

Noise‐related hearing and communication barriers were reported across all OR roles. Interventional studies are needed to assess the role of acoustic, cultural, and technological strategies for overcoming hearing barriers and promoting effective teamwork.

The operating room (OR) is one of the noisiest environments in healthcare, with average sound levels often exceeding 85 decibels and peaks reaching 130 decibels, far surpassing occupational safety thresholds.^1^, ^2^, ^3^, ^4^ This auditory environment reflects the aggregate effects of suction devices, alarms, drills, and other equipment, and overlapping conversations, creating an array of barriers to effective communication.^5^, ^6^, ^7^ In surgical settings where precision, vigilance, and responsiveness are essential, the contribution of noise to communication lapses can undermine patient safety, team performance, and surgical outcomes.^8^, ^9^, ^10^, ^11^

OR noise is distracting and an occupational hazard,^12^, ^13^, ^14^ with loud environments predisposing to noise‐induced hearing loss.^15^, ^16^, ^17^ However, this framing of noise as a physiologic exposure does not capture its role as a barrier to communication.^12^, ^18^, ^19^ Interruptions, alarms, and high ambient noise all contribute to communication failures. Furthermore, noise can degrade the learning environment, reducing ability to hear, understand, or speak up; noisy, hierarchical environments can thus reduce safety and educational opportunities.^20^, ^21^, ^22^, ^23^

This study addresses that gap by evaluating hearing and communication difficulties across the full spectrum of OR personnel at a large academic medical center. Using a mixed‐methods design, we quantified the prevalence of hearing challenges, examined associations with hearing status and occupational exposure, and analyzed qualitative responses to capture lived experiences and proposed solutions. By moving beyond measurements of noise to its impact on human communication, this study reframes OR noise as a systemic barrier—one that disproportionately affects certain roles, undermines training and teamwork, and demands multifaceted interventions in technology, behavior, and culture.^24^, ^25^, ^26^

## Methods

### Study Design

A cross‐sectional anonymous online survey was disseminated to OR personnel to assess noise‐related barriers to communication, the acoustic environment, and hearing. The survey captured respondent perspectives on difficulty with hearing in the OR, common sources of noise, and existing or potential solutions to improve communication. Demographic information was collected, allowing stratification of results by professional role, years of experience, and perceived hearing status. Findings were reported in accordance with the Checklist for Reporting Results of Internet E‐Surveys (CHERRIES).^27^

### Ethical Review

The study was approved by the Institutional Review Board of the University of Michigan (HUM00250841). Participation was voluntary and anonymous, and no identifiable information was collected.

### Survey Development

The survey was developed using a participatory design framework with interprofessional input from otolaryngology, general surgery, nursing, medical education, and electrical engineering. Survey development and refinement adhered to established best practices in survey methodology, with attention to avoiding ambiguity, leading phrasing, double‐barreled questions, and ordering effects.

The final survey instrument included 21 items and encompassed 4 domains: demographics, hearing status, communication barriers, and proposed solutions (Supplemental Table S1, available online). The survey was administered using Qualtrics software, version 2020 (Qualtrics), which enabled branching logic and randomized ordering to minimize bias.

### Participant Sample

The population sampled included all OR personnel involved in surgical services at a single academic center comprising surgeons, anesthesiologists, surgery and anesthesia residents, medical students, nurses, certified registered nurse anesthetists (CRNAs), scrub technicians, and allied health professionals. To be eligible, respondents were required to have active involvement in OR settings during the past 12 months. Demographic data were queried on professional role, years of OR experience, and self‐reported hearing status.

### Survey Administration

The survey was distributed once via institutional listservs to an estimated 1388 OR personnel. No follow‐up reminders were sent. Recruitment emails explained the study's purpose, time commitment, and confidentiality assurances, highlighting that the survey was voluntary, anonymous, and involved no identifying information. The survey remained open for 4 weeks, during which responses were collected through Qualtrics software (Version 2020; Qualtrics). Built‐in tools were used to verify completeness and prevent duplicate submissions through IP address tracking and session monitoring. Mandatory fields ensured all included responses were complete.

### Survey Questionnaire Content and Outcome Measures

The survey assessed auditory and communication challenges in the OR using multiple‐choice, Likert‐scale, and open‐text formats. Display logic ensured participants only viewed items relevant to their experience. Those who indicated “yes” or “maybe” for baseline hearing loss were asked additional questions about assistive device use, effectiveness, and context‐specific communication challenges. Hearing status was self‐reported and not audiometrically verified; respondents selecting “yes” indicated a prior clinical diagnosis, while “maybe” reflected subjective difficulty without a known abnormal audiogram. Questions queried sources of noise (eg, suction, alarms, drills, music, overlapping conversations), physical barriers (eg, masks, robotic consoles, or drapes), psychosocial barriers (eg, hierarchy, fear of negative evaluation, social stigma), strategies for coping with hearing difficulty (eg, repetition, anticipation, environmental adjustments, disclosure), and preferences for interventions (eg, noise reduction, behavioral interventions). The survey did not collect granular, procedure‐level case data (eg, specific orthopedic or otologic operations); respondents instead selected operative contexts they perceived as most challenging for communication. The primary outcome was the prevalence and frequency of self‐reported hearing difficulty in the OR. Secondary outcomes included associations with baseline hearing loss, years of OR experience, and role, as well as reported strategies and intervention preferences.

### Data Collection

Survey responses were stored securely on a password‐protected institutional server. Qualtrics functions were used to exclude duplicate entries, and mandatory fields ensured that all responses were complete.

### Statistical Analysis and Qualitative Synthesis

Quantitative analyses were conducted using IBM SPSS Statistics (version 22; IBM Corp.). Descriptive statistics summarized participant demographics and survey responses. Chi‐square and Fisher's exact tests were used to evaluate associations between categorical variables, such as professional role and hearing difficulty. *T*‐tests were used to compare group means, and correlation analyses explored associations between years of experience and perceived hearing difficulties.

Participants were stratified based on time spent in the OR, using a 5‐year cutoff to distinguish short‐term (<5 years) from long‐term (≥5 years) exposure. This threshold aligns with occupational health literature indicating that measurable effects of noise‐induced hearing loss often emerge after prolonged exposure.^12^, ^28^, ^29^ Given that OR noise frequently exceeds 70 to 80 dB, cumulative auditory risk was hypothesized to increase among those with longer exposure after adjusting for age.^2^, ^3^ To minimize confounding, responses of “maybe” regarding baseline hearing loss were excluded from regression models, leaving only “yes” and “no” responses. Logistic regression models included years of OR exposure, age, professional role, and baseline hearing loss status as covariates. Incomplete surveys were excluded, and no imputation was performed.

Qualitative responses from open‐text fields were analyzed using a summative approach to thematic analysis. Two independent (SEH and EYL) reviewers coded responses, identified recurring themes, and resolved discrepancies by consensus. A third reviewer (ASB) adjudicated discrepancies. Representative quotes were selected to illustrate barriers, coping strategies, and recommendations to ensure analytical rigor and capture the breadth of participant experiences.

## Results

### Participant Characteristics

A total of 225 OR personnel completed the survey (response rate, 16.2%). Respondents included medical students (n = 62, 27.6%), certified or registered nurses (n = 51, 22.6%), attending anesthesiologists (n = 33, 14.7%), CRNAs (n = 31, 13.8%), attending surgeons (n = 20, 8.8%), anesthesia residents/fellows (n = 17, 7.6%), and surgical residents (n = 11, 4.9%). Median age range was 31 to 35 years old. Median OR experience was 6 to 10 years, with 41% (92/225) reporting ≥10 years of OR exposure. Demographics are provided in Table 1. Mandatory survey fields ensured that all responses were complete, with no missing data across demographic or role‐specific questions.

**Table 1:** Respondent Demographics

Characteristic	n (%)
Participant demographics	
Total participants	225 (16%)
Surgical attendings	20 (9%)
Anesthesia attendings	33 (15%)
Surgical residents	11 (5%)
Anesthesia residents	17 (8%)
Nurses	51 (23%)
Medical students	62 (28%)
CRNAs	31 (14%)
Age range (median)	31‐35
Age distribution	
<50 years	163 (72%)
>50 years	62 (28%)
Years of OR experience (Median)	6‐10
Distribution of OR experience	
<2 years	81 (36%)
2‐5 years	26 (12%)
6‐10 years	31 (14%)
11‐20 years	56 (25%)
>20 years	0 (0)
Hearing status	
No baseline hearing loss	135 (60%)
Baseline hearing loss	24 (11%),
“Maybe” Group	66 (29%)

### Hearing status in respondents

Among 225 respondents, 11% (24/225) reported diagnosed hearing loss, 60% (135/225) reported no awareness of hearing loss, and 29% (66/225) had suspected hearing loss. Respondents in the suspected category had no history of abnormal audiogram and self‐described as “hearing, but occasionally unsure.” Individuals in this group might have frequent subjective difficulty with hearing but no definitive diagnosis of hearing loss and were designated (“Maybe”).

### OR hearing challenges across all roles

**Figure 1:**
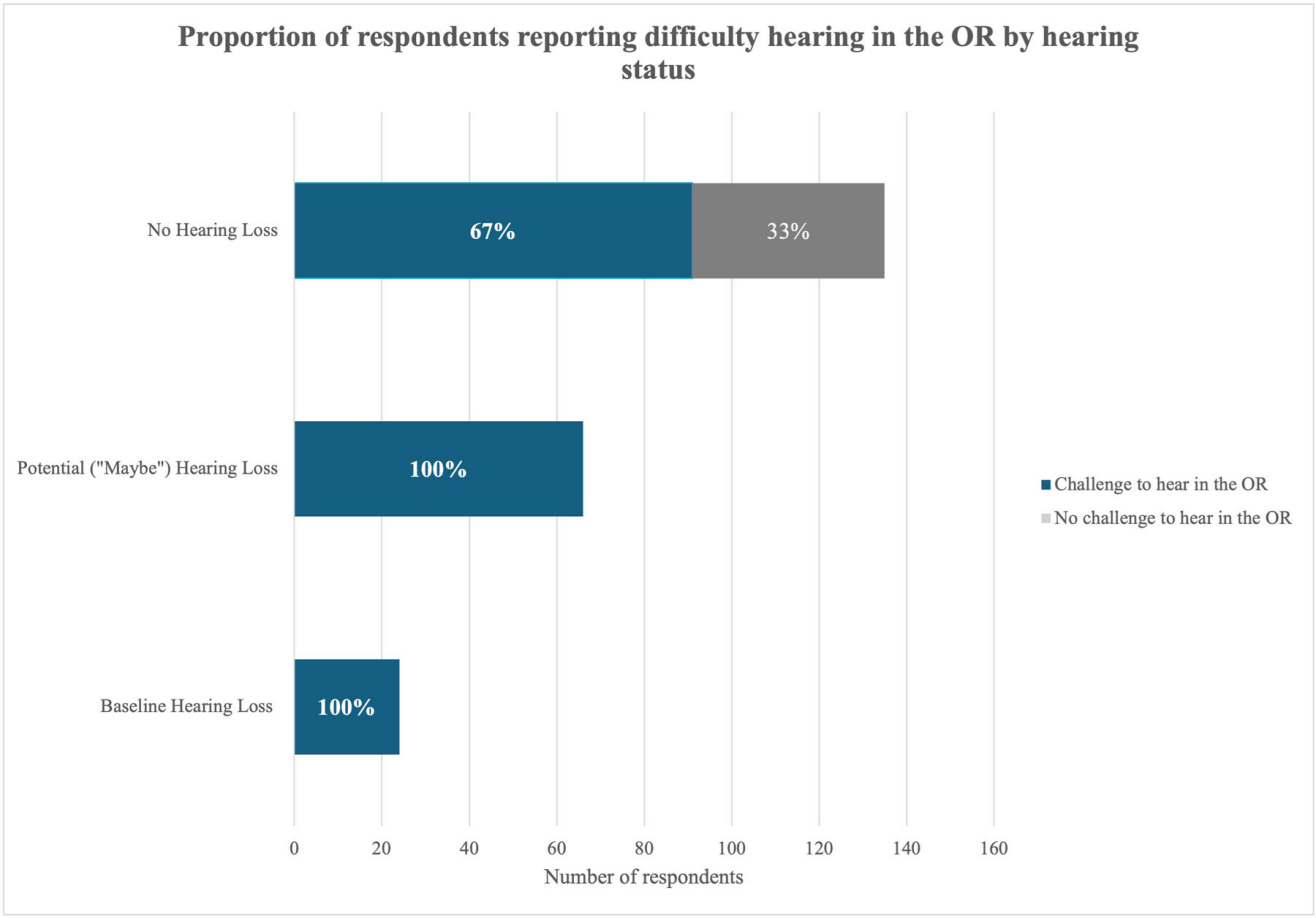
Self‐reported hearing difficulty in the operating room by hearing status. Proportion of respondents reporting difficulty hearing in the OR, stratified by self‐reported hearing status.

The distribution of respondents struggling to hear in the OR, stratified by Hearing Loss category (ie, no baseline hearing loss, possible baseline hearing loss but unsure, and confirmed baseline hearing loss) is summarized in Figure 1.

#### No Baseline Hearing Loss

Among the 135 (60%, 135/225) with no baseline hearing loss, 67% (91/135) reported challenges hearing in the OR at a frequency of every case (11%, 10/91), daily (35%, 32/91), weekly (32%, 29/91), and monthly (22%, 20/91).

#### Possible Hearing Loss

Of 66 respondents who identified “hearing, but occasionally unsure” due to frequent issues with hearing, 100% (66/66) reported difficulty hearing in the OR. At a frequency of every case (14%, 9/66), daily (18%, 12/66), weekly (30%, 20/66), and monthly (38%, 25/66).

#### Established Baseline Hearing Loss

All 24 respondents (100%, 24/24) with baseline hearing loss reported struggling to hear in the OR at least sometimes, most reporting difficulty hearing every case (58%, 14/24).

### Hearing Loss Sub‐Group

When analyzed by profession, 53% (13/24) of respondents who reported baseline hearing loss were nurses, 12.5% (4/24) were medical students, 21% (5/24) were attending surgeons, 4% were resident surgeons (1/24), and 4% (1/24) were attending anesthesiologists.

To explore whether hearing loss or difficulty hearing in the OR was related to age, participants were grouped into 2 categories: under 50 years old and over 50 years old. This cutoff aligns with clinical benchmarks for age‐related hearing loss, or presbycusis,

which typically begins to manifest around age 50.^30^, ^31^, ^32^ No significant association was found between age group and hearing loss (*P* = .89) or age and hearing difficulty among those without diagnosed hearing loss (*P* = .92). Among the 24 respondents with baseline hearing loss, 67% (16/24) report daily hearing aid use, and 88% (14/16) of hearing aid users found it challenging to understand speech in the OR. All respondents with hearing aids indicated that their devices were appropriately fitted and they did not have issues in unmasked, less complex acoustic environments. We collected data on daily hearing aid use but did not query whether this daily use occurred in the OR. Respondents reporting daily use were asked to reflect on their communication experiences in the OR context. One respondent shared, “Even with hearing aids, I have to constantly ask people to repeat themselves or speak louder. It's very frustrating.”

### Hearing Loss and Occupational Exposure Duration

Among all respondents, 39% (88/225) indicated concern about occupational noise exposure from the OR environment. Among the 24 participants who reported hearing loss, 25% (6/24) had <5 years of operating room experience, while 75% (18/24) had ≥5 years (*P* = .023). After adjustment for age, professional role, and baseline hearing loss status, individuals with ≥5 years of OR exposure had 3.0 times higher odds of reporting baseline hearing loss compared to those with <5 years (95% CI: 1.2‐7.9, *P* = .024).

### Communication Barriers in the OR

Qualitative analysis yielded 3 overarching themes: (1) environmental and physical barriers to communication, (2) psychosocial barriers and power dynamics, and (3) adaptive strategies and desired interventions. All themes and subthemes are summarized in Table 2 and illustrated below with representative quotations and contextual examples.

**Table 2:** Key Themes and Sub‐Themes Identified From Free‐Text Survey Responses

Key Theme	Sub‐Theme	Representative Comments
Hierarchy and Psychological Safety	Reluctance to speak up	“As a student, I just keep quiet when I miss something — it doesn't feel safe to ask again.” (*Third‐year medical student*) “Residents hesitate to stop the case to clarify when the attending isn't clear.” (*Surgical resident*)
	Power dynamics	“It's intimidating to interrupt a senior surgeon to say you didn't hear.” (*OR nurse*) “You risk being seen as someone who can't keep up.” (*Anesthesia resident*)
Environmental Noise Sources	Competing equipment	“Drills and suction at the same time completely block voices.” (*Surgical tech*) “Anesthesia machine alarms overwhelm everything else.” (*CRNA*)
	Music and overlapping talk	“Loud music makes it difficult to follow instructions.” (*Scrub technician*) “Multiple conversations at once make it hard to know who to listen to.” (*OR nurse*)
Communication Modality Barriers	Masks and muffling	“With masks, I lose lip reading — half the information is gone.” (*Second‐year medical student*) “Muffled voices make it hard to catch the words or tone.” (*Surgical resident*)
	Distance and robotics	“When the surgeon is at the console, their voice doesn't carry.” (*Resident surgeon*) “Across the drapes, I only catch part of the message unless repeated.” (*OR nurse*)
Variability in Speech	Soft‐spoken team members	“Soft‐spoken staff are almost impossible to hear in the OR.” (*Surgical attending*) “If someone doesn't project their voice, it gets lost in the noise.” (*OR nurse*)

Respondents also identified barriers unrelated to procedure type or specialty. Many respondents pointed to overlapping conversations and background equipment noise as challenges. One surgical resident explained, “It feels like three people are talking at once, and I can't catch who's speaking to me.” Others noted that communication was more difficult when speakers were not facing them, with a medical student reporting, “When someone talks while facing away from me, I miss half of what they said.” Several respondents described hesitancy to ask for clarification, with one respondent stating, “Asking for repetition in the middle of a case can feel disruptive.” The effects of noise on communication were also noted to differ as a function of the type of noise and sound quality of the speaker's voice. For example, one anesthesiologist remarked, “The monitor beeps drown out the softer voices*.*” (Table 2).

### Communication Challenges in Specific OR Environments

Respondents identified several communication challenges related to specific OR contexts. Orthopedic cases were most frequently mentioned (31%, 70/225), with participants reporting noise from hoods, drills, and other power tools such as suction irrigators. Environmental noise, including suctioning and music, was highlighted by 29% (65/225) of respondents. Robotic cases were described as difficult by 24% (55/225), primarily due to muffled voices and the distance created by the surgeon working at the console. Additional challenges included crowded rooms and side conversations (15%, 33/225).

### Approaches to Self‐Accommodation in the OR Across Roles

Respondents described a range of self‐accommodation strategies to manage hearing and communication challenges in the OR, including repetition, anticipation, repositioning, environmental modification, and disclosure. These approaches are summarized in Table 3 and elaborated below with representative quotations and examples.

**Table 3:** Qualitative Analysis of Approaches to Self‐Accommodation in the OR

Key theme	Representative comments
Repetition and Clarification	“I ask people to repeat themselves, but after the second time it feels uncomfortable.” (*OR nurse*) “If I miss a dose confirmation, I immediately ask again to be safe.” (*CRNA*)
Anticipation of Instructions	“I try to predict what's coming next so I don't miss something important.” (*Third‐year medical student*) “Following the steps of the case helps me fill in gaps when I don't hear clearly.” (*Surgical resident*)
Environmental Adjustments	“If possible, I turn down the music or lower the alarm volume.” (*OR nurse*) “I'll ask for suction to be clamped when it's not being used.” (*CRNA*)
Positioning and Proximity	“I move closer to the field if I'm struggling to hear.” (*OR nurse*) “Leaning in slightly makes a big difference with soft‐spoken colleagues.” (*OR nurse*)
Role‐Specific Adaptations	“As a student, I stay quiet and watch carefully rather than asking repeatedly.” (*Second‐year medical student*) “Residents often rely on context instead of asking again.” (*Anesthesia resident*)
Disclosure and Coping	“I've mentioned my hearing trouble once or twice, but I usually keep it to myself.” (*Surgical attending*) “I avoid disclosing because I don't want it to be seen as a weakness.” (*Surgical resident*)

Participants across roles described a range of strategies to adapt to hearing challenges in the OR. The most reported approach was asking for repetition or clarification (58%, 131/225). A resident noted, “I'll usually ask once, but after that I just try to follow along as best I can.” Anticipating instructions or next steps was described by 36% (81/225), with a medical student sharing, “If I don't hear something, I just stay quiet and try not to look confused.” Environmental adjustments, such as lowering music or changing monitor settings, were reported by 28% (64/225). Improving proximity or positioning was cited by 20% (46/225), with one nurse explaining, “If I can move closer to the field, it's a lot easier to hear.” (Table 3).

Role‐specific strategies also emerged. Attending surgeons or anesthesiologists sometimes described deliberately repeating themselves or speaking louder for the team. Nurses often emphasized anticipating surgeon preferences or relying on colleagues for confirmation. Residents mentioned using context or visual cues to avoid interrupting the flow of the case. Medical students, in contrast, frequently described a reluctance to ask for clarification, with one commenting, “I don't want to be the person holding things up, so I just stay quiet and hope I guessed right.” Another remarked, “Sometimes I'm completely lost, but it feels too intimidating to admit I didn't hear.” Disclosing hearing loss or requesting written instructions was the least common strategy (5%, 12/225), with respondents acknowledging discomfort. One respondent wrote, “It feels risky to admit I can't always hear everything.”

### Recommendations for Improving Communication and Accessibility in the OR

Nearly all respondents (94%, 212/225) indicated that communication in the context of ambient noise could be improved in the OR. The most frequently mentioned strategy was reducing noise and improving acoustics (38%, 86/225). Suggestions included lowering or turning off music, clamping suction when not in use, and dampening noise from drills or other equipment. One participant remarked, “The background noise makes even simple instructions difficult to hear.”

Improved communication practices were recommended by 27% (61/225), including speaking louder, avoiding mumbling, and using clear, concise language. Several respondents also endorsed structured approaches such as closed‐loop communication to ensure clarity. A resident explained, “Repeating back what was said helps everyone, not just the person who missed it the first time.”

Assistive tools were identified by 18% (40/225) of participants. Proposed solutions included clear masks, microphones, and amplification devices to help voices carry over noise. A medical student noted, “Even basic captioning or microphone use would make a huge difference.”

Environmental adjustments were less frequently cited (9%, 21/225) but included improving room layouts and addressing equipment placement to minimize interference. Training on communication strategies was suggested by 7% (15/225), particularly for orienting new learners or ensuring consistency across teams.

## Discussion

In our sample of OR personnel, hearing and communication difficulties were common, with over 60% reporting problems and nearly 1 in 10 noting challenges in every case. Even those without diagnosed hearing loss frequently struggled in high‐noise environments, underscoring that this is a shared concern. The three‐fold higher odds of hearing loss among individuals with ≥5 years of OR exposure, independent of age, suggests cumulative occupational risk. Drawing on an interprofessional sample of surgeons, anesthesiologists, trainees, nurses, CRNAs, and scrub technicians, the mixed‐methods design integrated quantitative and qualitative insights, highlighting the pervasiveness of noise‐related communication barriers in the OR.

These findings align with and expand on prior literature in this area. Continuous and intermittent acoustic sources (suction, powered instruments, alarms, overlapping conversations, and music) create unfavorable signal‐to‐noise ratios for understanding speech.^1^, ^3^, ^5^ Communication across drapes, within consoles, or with the person speaking while facing away from the recipient further exacerbate the problem by removing visual and contextual cues such as facial expression, lip movement, and subtle gestures that many individuals rely on unconsciously to supplement auditory input. Qualitative responses highlighted the consequences of these dynamics: uncertainty, reliance on repetition, and hesitancy to ask for clarification in hierarchical environments. Learners described “staying quiet” or “guessing” rather than risking interruption, reflecting how psychological safety intersects with auditory accessibility.^8^, ^20^, ^21^, ^23^ These findings suggest a need to expand the conceptualization of OR noise beyond an environmental nuisance or exposure, toward a systems‐level barrier that shapes not only audibility but also attention and cognitive load in high‐stakes environments.^12^, ^15^ Reframing noise as a barrier to equitable participation and safe communication where missed information may reflect divided or redirected attention rather than volume alone is consistent with prior work demonstrating the deleterious effects of distractions.^7^, ^10^, ^33^

Respondents described compensatory behaviors such as repetition, anticipation, repositioning, and environmental modification.^9^, ^10^ While adaptive, these strategies increase cognitive load and fatigue. The most frequent recommendations were acoustic hygiene measures (eg, lowering music, clamping suction, adjusting alarms)^11^, ^13^ and improved communication practices (clearer speech, structured closed‐loop confirmation).^34^, ^35^, ^36^ Assistive strategies such as microphones, amplification, and clear masks were less commonly reported but hold promise as technology for real‐time speech enhancement and captioning advances.^19^, ^26^ Environmental adjustments (room layout, equipment positioning) and team training were also cited as potential supports.

These findings have implications for both patient safety and surgical education. Communication errors can lead to errors in communication handoffs, dose confirmations, or key procedural steps, and background noise or muffled speech can have downstream effects that are difficult to measure. For trainees, difficulty hearing instructions limits opportunities for learning and participation, reinforcing hierarchies and constraining psychological safety.^25^, ^37^, ^38^ For perioperative staff, prolonged reliance on compensatory strategies is fatiguing and inefficient. Coordinated efforts are needed at multiple levels: (1) environmental and acoustic interventions, (2) behavioral practices that normalize repetition and closed‐loop communication, and (3) technology‐enhanced solutions to improve audibility without increasing cognitive burden.

This study has several limitations. First, it used a cross‐sectional survey design conducted at a single institution, which limits generalizability. Second, responses were based on self‐reported perceptions of hearing loss and noise exposure rather than objective measurements. Because hearing status was self‐reported and not verified with audiometry, this study cannot determine the degree of concordance between perceived and objective hearing loss in this cohort. Third, the study lacked longitudinal assessment and may be subject to selection, nonresponse, and recall bias. The survey was distributed once without reminder emails, which may have limited participation and contributed to nonresponse bias; future studies should incorporate follow‐up reminders to enhance response rates. Finally, the survey did not capture procedure‐specific case types, limiting the ability to compare communication barriers across individual surgical procedures or specialties. Despite these constraints, the findings provide novel insights and a rationale for future investigation and efforts to promote effective communication in the OR.

Future research should expand to multi‐institutional cohorts and integrate objective measures of noise exposure, audiometry, communication accuracy, and procedure‐specific case data to more precisely characterize how acoustic environments and operative context shape hearing and teamwork in the OR.^28^, ^29^, ^39^ In addition, studies are needed to understand the outcomes of pragmatic intervention bundles that combine acoustic hygiene, structured communication practices, and technology augmentation.^40^, ^41^ In parallel, institutions can create pathways for confidential screening, accommodations, and education to promote the effective communication necessary for surgical safety and optimal team performance.^42^, ^43^

## Conclusion

Hearing and communication challenges in the OR are frequent, role‐spanning, and consequential. The combination of excessive noise and communication breakdowns undermines both psychological safety and patient safety, with downstream effects on teamwork, learning, and clinical outcomes. Solutions must therefore be multifaceted—embedding accessibility and acoustic awareness into the culture, environment, and practices of surgical teams. Addressing these barriers is a matter of inclusion and an essential component of safe and effective surgical care.

## Author Contributions

**Sarah E. Hughes**, conceptualization, design, methodology, investigation, data curation, formal analysis, writing—original draft; **Milisa Manojlovich**, methodology, writing—review and editing; **Eunice Y. Lee**, investigation, data analysis, data curation, writing—review and editing; **Andrew S. Bolze**, data analysis, data curation, writing—review and editing; **Candice Stegink**, project administration, data curation, writing—review and editing; **Dhruv Jain**, methodology, resources, writing—review and editing; **Devin L. McCaslin**, conceptualization, supervision, writing—review and editing; **Kyle H. Sheetz**, methodology, validation, writing—review and editing; **Rishindra M. Reddy**, supervision, resources, writing—review and editing; **Michael J. Brenner**, methodology, supervision, data interpretation, writing—original draft, writing—review and editing, funding acquisition.

## Disclosures

### Competing interests

None.

### Funding source

This study was supported by the NIH R25 grant (5R25DC20262‐02) awarded to SH through the Michigan Otolaryngology Research Education (MORE) program.

## Supporting information

Supplemental Table 1. Survey Instrument.
Complete 21‐item survey used in this study, including wording, response options, and branching logic across the four domains of demographics, hearing status, communication barriers, and proposed solutions.
