# Supplementary material for: Noise, Hearing, and Communication in the Operating Room: A Mixed‐Methods Study
Source: Otolaryngol Head Neck Surg. 2026 Apr 30;175(2):364–72. doi: 10.1002/ohn.70266 (PMC13417945; doi:10.1002/ohn.70266)
Supplement: Supplementary file 1 — Supplemental Table 1. Survey Instrument. Complete 21‐item survey used in this study, including wording, response options, and branching logic across the four domains of demographics, hearing status, communication barriers, and proposed solutions. [file OHN-175-364-s001.docx]

**Supplemental Table 1. Survey Instrument**

| Domain | Item # | Survey Question | Response Options |
| --- | --- | --- | --- |
| Demographics | 1 | Q1. What is your role in the Operating Room? | Circulating nurse, Scrub tech, CRNA, Resident surgeon, Anesthesia resident/fellow, Attending surgeon, Attending anesthesiologist, Medical student, First assistant, Other |
|  | 2 | Q2. How many years have you worked in an OR? | <2, 2–5, 6–10, 11–20, >20 |
|  | 3 | Q3. How old are you? | 18–25, 26–30, 31–35, 36–40, 41–45, 46–50, 51–55, 56–60, 61–65, 66–70, >70 |
| Hearing Status | 4 | Q4. Do you have baseline hearing loss? | Yes / No / Maybe |
|  | 5 | Q5. Do you have difficulty hearing in the OR? | Yes / No |
|  | 6 | Q5a. How often do you have difficulty hearing in the OR? | Every case, Daily, Weekly, Monthly, Yearly, Never |
|  | 7 | Q8. Has difficulty hearing affected your career plans? | Yes / No |
|  | 8 | Q11. Do you wear hearing aids in the OR? | Yes / No |
|  | 9 | Q11a. Do you find find it challenging to hear in the OR even when utilizing hearing aids? | Yes / No (with optional free text) |
|  | 10 | Q12. Do you use other assistive devices or strategies in the OR? | Yes / No (with optional free text) |
| Communication Barriers | 11 | Q9. How often do you have difficulty hearing in the OR? | Every case, Daily, Weekly, Monthly, Yearly, Never |
|  | 12 | Q10. Do you find the use of masks in the OR to be a communication barrier? | Yes / No |
|  | 13 | Q11. Which sources of noise are most problematic? | Suction, Drills, Alarms, Music, Overlapping conversations |
|  | 14 | Q12. Which strategies do you use to cope with hearing difficulty? | Ask repetition, Move closer, Anticipate, Adjust equipment, Other (free text) |
|  | 15 | Q13. Do you feel communication could be improved in the OR? | Yes / No |
| Perceptions & Solutions | 16 | Q14. Is there a service or procedure where communication is especially challenging? | Free text (examples: laparoscopy, robotic, orthopedic drill cases) |
|  | 17 | Q15. How do you think the OR could be a more accessible environment for people with hearing loss? | Free text |
|  | 18 | Q16. Anything else you would like to comment on regarding communication and acoustics in the OR? | Free text |
|  | 19 | Q17. Are you concerned about occupational noise exposure hearing loss from time spent in the operating room? | Yes / No |
|  | 20-21 | (Embedded follow-ups from Q5 - Q13: elaborations on coping strategies, mask barriers, noise sources, and proposed solutions) | Open-text fields |
